# Hypohidrotic Ectodermal Dysplasia: A Felicitous Approach to Esthetic and Prosthetic Management

**DOI:** 10.5005/jp-journals-10005-1207

**Published:** 2013-08-26

**Authors:** Tapan Singh, Ronauk Singh, Gurendra Pal Singh, Jitender pal Singh

**Affiliations:** Postgraduate Student, Department of Pedodontics, Saraswati Dental College, Lucknow, Uttar Pradesh, India, e-mail: tapandr@in.com; Captain, Department of Prosthodontics, Army Dental Corps, West Bengal, India; Professor, Department of Periodontics, Saraswati Dental College Lucknow, Uttar Pradesh, India; Major General, Department of Prosthodontics, Army Dental Corps Maharashtra, India

**Keywords:** Ectodermal dysplasia, Anodontia, Prosthodontic rehabilitation

## Abstract

Ectodermal dysplasia is a hereditary disease characterized by congenital dysplasia of one or more ectodermal structure and other accessory appendages. The oral manifestations are anodontia and poor bony foundation which impairs both esthetic as well as the masticatory function. The prosthodontic management of patients with such dysplastic condition necessitates a multidisciplinary approach. This case report describes the prosthodontic oral rehabilitation of a 16 years old female pediatric patient with ectodermal dysplasia.

**How to cite this article:** Singh T, Singh R, Singh GP, Singh JP. Hypohidrotic Ectodermal Dysplasia: A Felicitous Approach to Esthetic and Prosthetic Management. Int J Clin Pediatr Dent 2013;6(2):140-145.

## INTRODUCTION

The ectodermal dysplasias (ED) are a heterogeneous group of over 150 syndromes characterized by disturbances in the formation and function of ectodermally derived structures, mainly hair, nails, teeth and sweat glands. Dental findings may consist of total anodontia of either the primary or permanent teeth. Cone-shaped teeth frequently occur. The maxilla and mandible grow independently and develop to normal size and shape. The absence of salivary glands is not a common finding and most patients do not complain of xerostomia. Earlier reports^1-3^ have described the most common type of ED as anhidrotic ED because of the lack of sweat gland function. Due to partial absence of sweat and sebaceous glands, the phenotype has smooth, soft dry and thin skin. Fine linear wrinkles and increase pigmentation are often present around the eyes and mouth. The mode of inheritance of hypohidrotic ED can be either autosomal dominant (AD), autosomal recessive (AR) or X-linked recessive (XLR) and the gene locus is X q13-q21. It is commonly XLR with full expression in males; female carriers have a minimal expression in 60 to 70% of cases and usually show mild manifestation restricted to minimal hypodontia such as absent maxillary lateral incisors or presence of conical teeth.^4^ All racial groups have been afflicted by this condition. Attempts have been made in the past to develop objective diagnostic criteria based on the number and distribution of sweat pores and the number of sweat produced. Structural and biochemical characteristic of the hairs, dermatologly pic analysis, characteristics of the lacrimal secretion and the distribution and pattern of scalp hair have also been used. However, universally acceptable standards have not been provided.^5^ An important factor is that the facial appearance of affected individuals is so characteristic that unrelated patients may be mistaken for siblings.

## CLINICAL REPORT

**Figs 1A and B:**
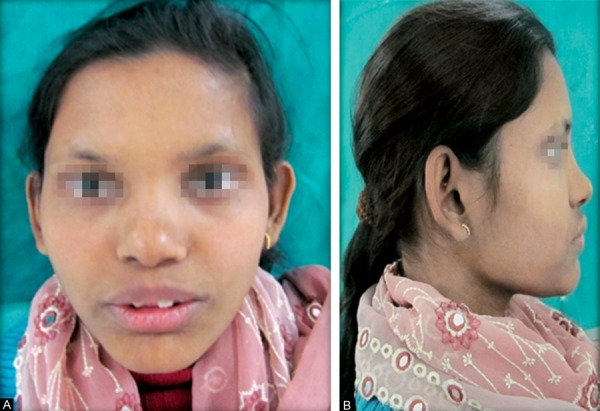
Facial features

**Fig. 2:**
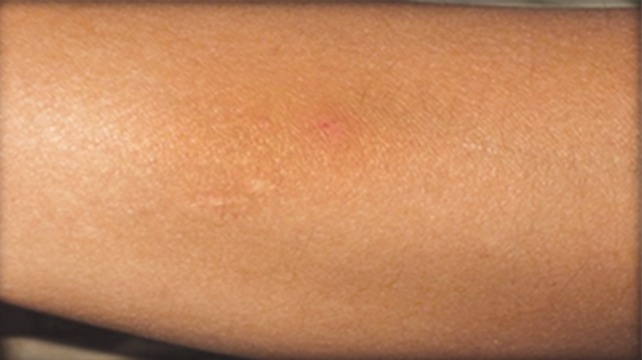
Dry skin feature, sparse hair on skin

**Fig. 3:**
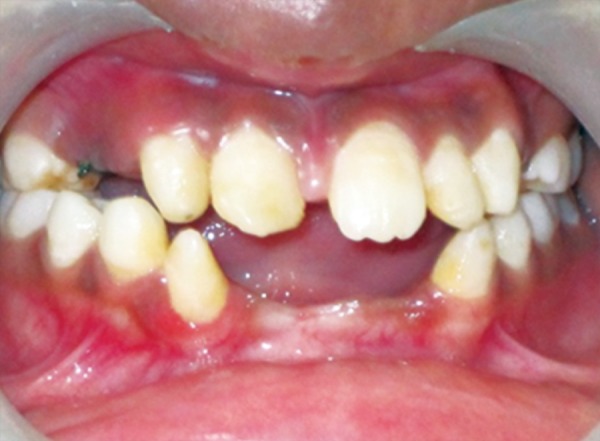
Intraoral photograph

**Fig. 4:**
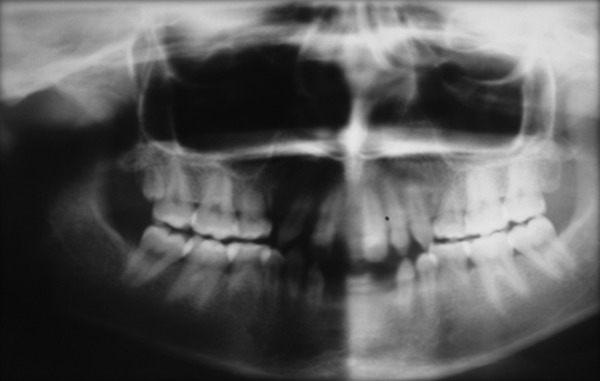
OPG shows mixed dentition

**Fig. 5:**
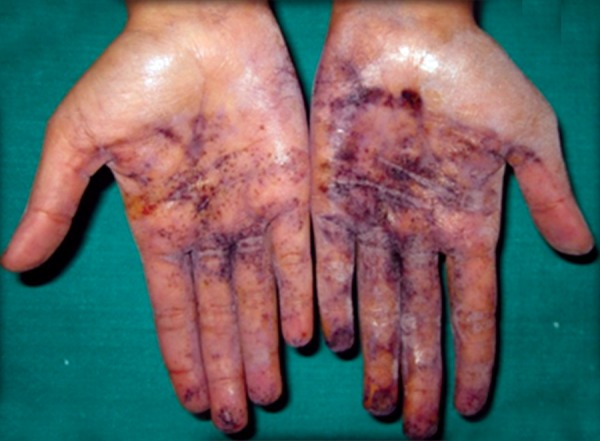
Starch iodine test shows scarce sebaceous glands

**Fig. 6:**
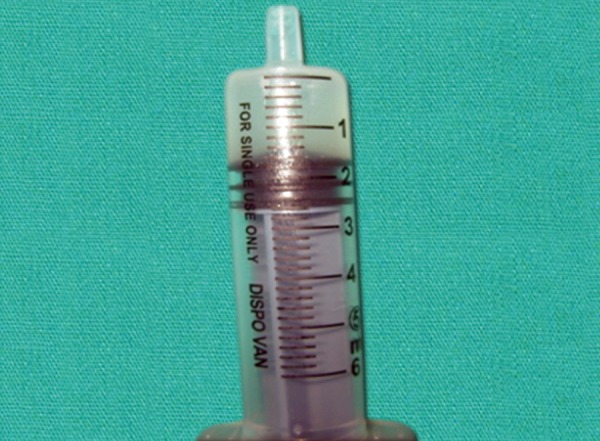
Amount of salivary secretion in a minute

**Fig. 7:**
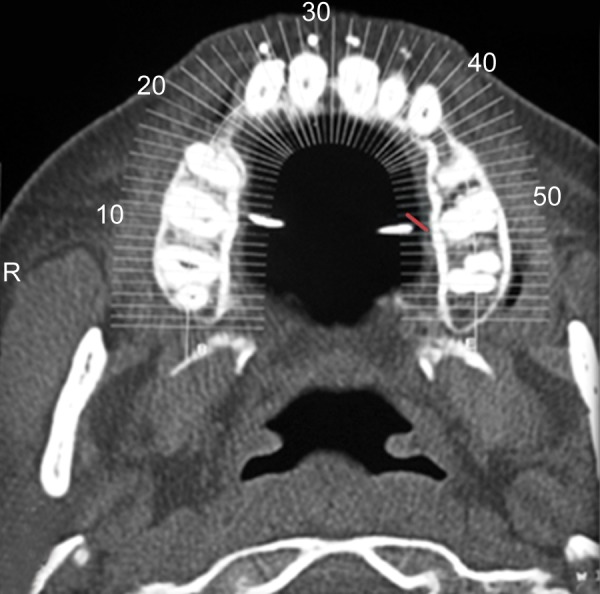
Dentascan image showing reduced bone width in maxilla

**Fig. 8:**
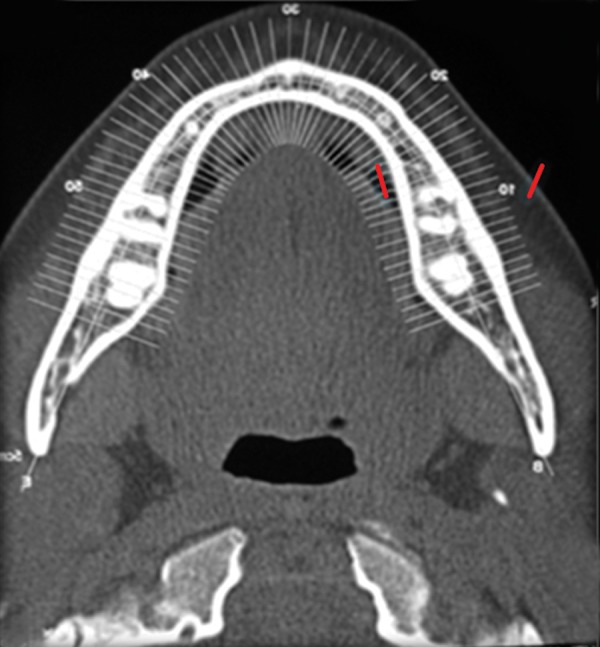
Dentascan image showing reduced bone width in mandible

**Fig. 9:**
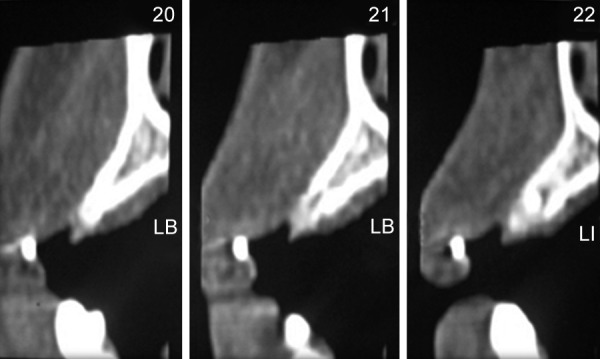
Area of interest with less bone thickness

**Fig. 10:**
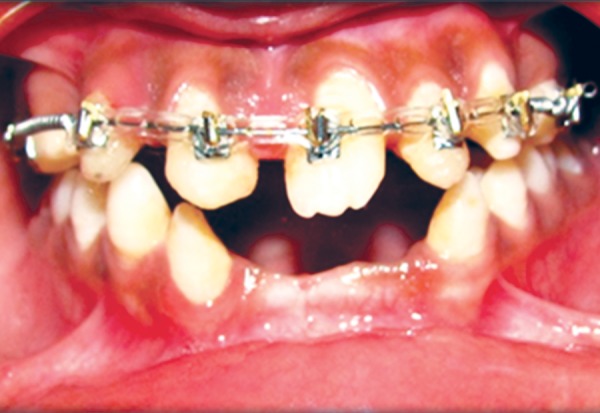
Preoperative orthodontic treatment

**Fig. 11:**
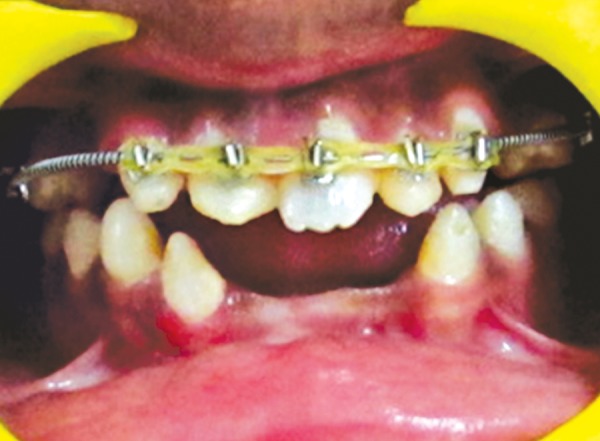
Postoperative orthodontic treatment

**Fig. 12:**
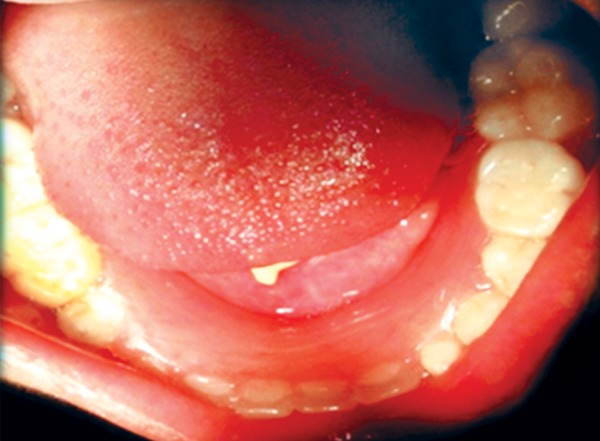
Retention phase removable appliance in maxilla

**Fig. 13:**
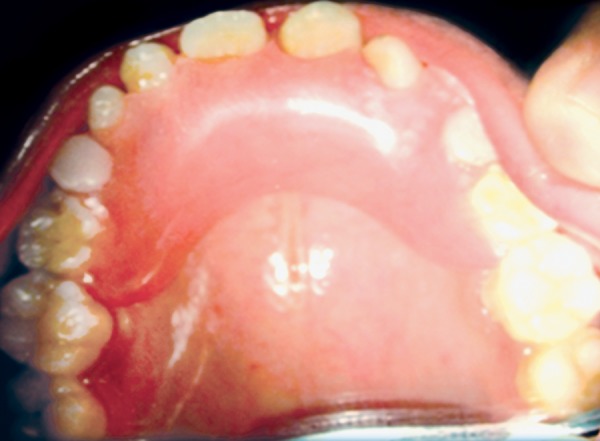
Retention phase removable appliance in mandible

**Fig. 14:**
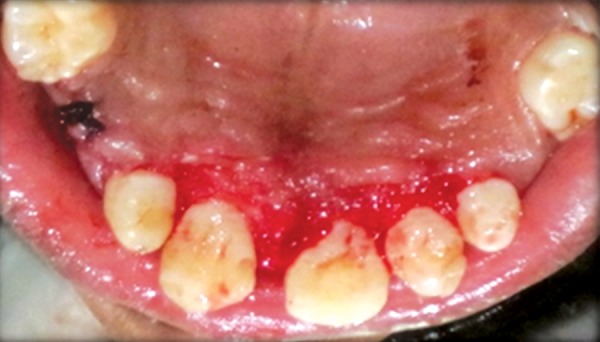
Gingivoplasty

**Fig. 15:**
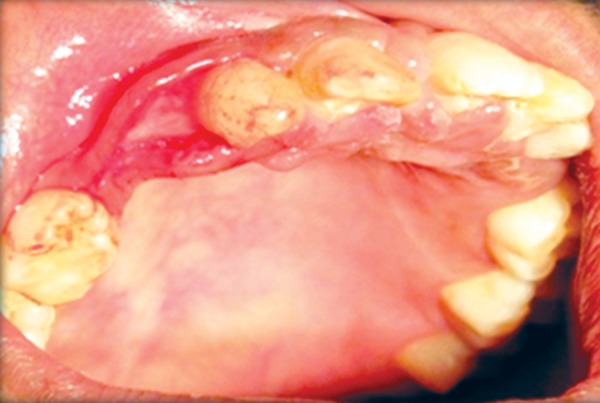
Alveoloplasty

**Fig. 16:**
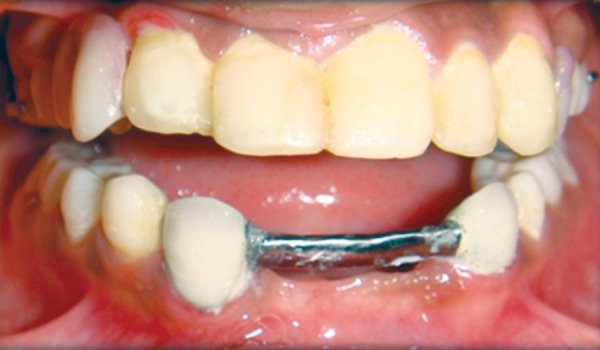
Direct composite laminates in relation to maxillary anterior and bar clasp in relation to mandibular anterior

**Fig. 17:**
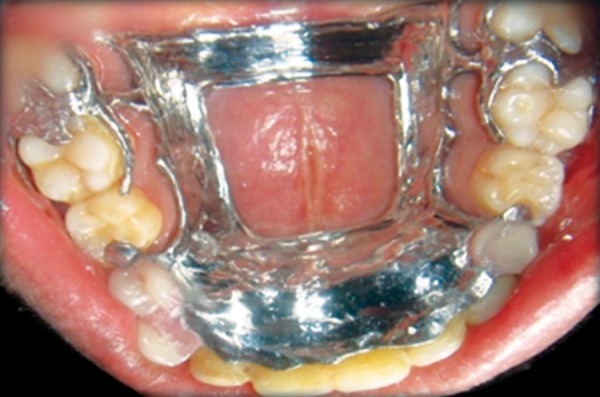
Cast partial denture irt maxilla

**Fig. 18:**
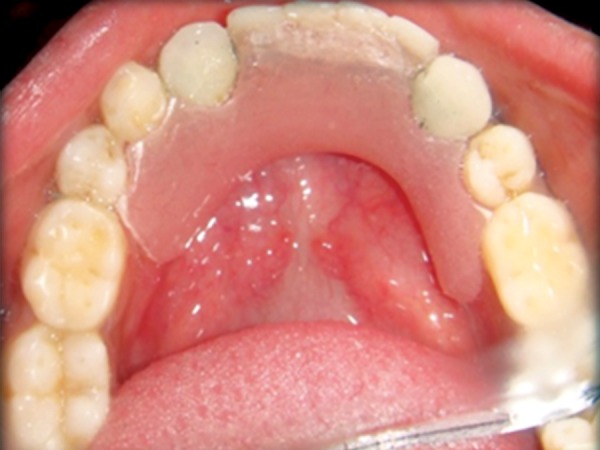
Intraoral view of overdenture

**Fig. 19:**
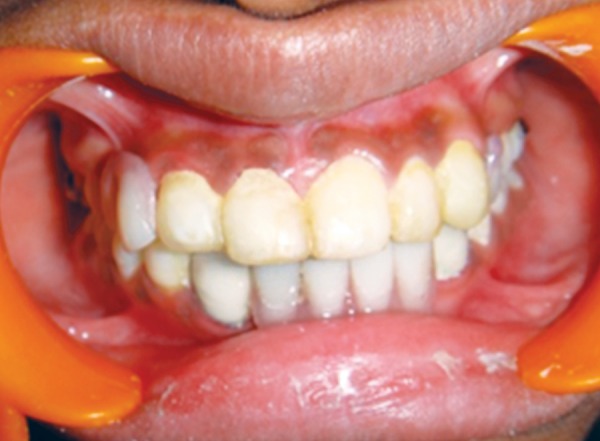
Postoperative photograph

The patient was a 16-year-old girl. His dental history included oligodontia with a mixed dentition of primary and secondary teeth. Clinical examination extraorally gave following features: (1) Prominent supraorbital ridge, (2) depressed nasal bridge, (3) sparse eyebrows, (4) protruded lips, (5) prominent and oblique set of ears (Fig. 1A and B). Skin features: (1) Skin over the entire body is dry, finely wrinkled and (2) hypopigmented; (3) there is sparse hair on the arm (Fig. 2). On intraoral examination the patient showed presence of (1) oligodontia, (2) teeth present have abnormal crown form, (3) teeth in the anterior region of maxilla and mandible are conical, (4) there is wide midline diastema, (5) retained deciduous teeth (Fig. 3). Teeth present were 8 permanent teeth in the maxilla (bilateral first and second molars, bilateral canine, unilateral central incisors and canine) and 5 deciduous teeth (canines and second molars). Mandibular showed 6 permanent teeth (mandibular first and second permanent molars and canine) and 5 deciduous teeth (second molar, canine and unilateral lateral incisor) (Fig. 4). In addition, the patient had an undeveloped mandibular alveolar ridge (knife ridge) (see Fig. 3). A panoramic radiograph revealed no development of primary or secondary teeth/tooth germs (see Fig. 4). Before starting with the treatment proper different tests were done to justify our action (1) starch iodine test using starch iodine powder was done to check for sebaceous gland on palm, which revealed that it was less (hypohidrotic ED) (Fig. 5). We did think implant as an option for the treatment but due negative result in salivary flow test, patient was advised to chew on paraffin wax for 1 minute and then saliva was collected (xerostomic) (Fig. 6) implant is contraindicated. Dentascan was also done which showed us that the bone thickness was to less to support the implant in both maxillary and mandibular arches (Fig. 7 and 8), region 20 and 21 (Fig. 9). Treatment was divided in 4 stages. Stage 1 oral prophylaxis and orthodontic treatment was done. After completion of orthodontic treatment, provisional splinting and prosthesis were given for maintenance and evaluation of response to etiotropic phase was done (Fig. 10 to 13). Stage 2 was surgical phase where gingivoplasty was done in relation to maxillary anterior and alveoloplasty in relation to 14 and 15 region (Fig. 14 and 15). Stage 3 was restorative phase, where direct composite laminates were given in relation to maxillary anteriors and bar clasp attachment was give in relation mandibular anterior with ceramic coping for overdenture (Fig. 16). Occlusal rest were prepared on tooth and after seating of the coping, rubber base impression was made for upper and lower arch, before making an impression for lower arch the undercut below the bar clasp was covered with modeling wax. Cast partial denture was fabricated in relation to maxillary arch and overdenture was given in relation to mandibular anterior (Fig. 17 to 19). Stage 4 maintenance phase which included periodic rechecking of plaque and calculus, gingival condition (pockets, inflammation), occlusion, tooth mobility.

## DISCUSSION

The hypohidrotic ED, otherwise called Christ-Siemens-Touraine syndrome (X–linked form) described by Thurnam in 1848.^6^ The ED is a group of inherited disorder that share in common developmental defects involving at least two of the major structure classically hold to derive from the embryonic ectoderms–hair, teeth, nails, sweat glands. Orally, the disease is characterized by hypodontia, oligodontia or anodontia, which can, moreover, affect both the maxilla and mandible, delayed eruption, malformed teeth producing a small, pointed conical appearance and resorption or atrophy of the alveolar border, thus complicating the fundamental rehabilitation procedure in these patients. Treatment of a child with ED requires a multidisciplinary approach and knowledge of behavioral management of the pediatric patient. The ED child can be rehabilitated using composite buildup, crowns, fixed prosthesis, overdentures, removable prosthesis or implants depending on the dentition present. Treatment should be aimed at maintaining the dentition present and alveolar ridges as these structures have to support the denture prosthesis for a life time. The prosthetic treatment should be carried out on an individual basis, aimed always toward providing good occlusal stability. It also aids in phonation and mastication. These factors instill greater self confidence in the child and help him gain acceptance. ED is usually a difficult condition to treat with prosthodontic restorations because of the typical oral deficiencies and the young age when they are evaluated for treatment. Therefore, when treating a child with ED, it is important to motivate both the child as well as his parents prior to the treatment and to work with them to ensure their compliance. It is a multidisciplinary approach. Literature shows that children rejected by their peer groups are more likely to become aggressive, delinquent, and may experience mental health problems in adulthood.^7^ Therefore, successful treatment of the present case can be expected to assist the patient both physically and psychologically. The boy's attitude, self confidence, and peer group interaction showed signs of significant improvement during treatment. Clinical reports have stated the importance of prosthetic dental treatment in patients with anodontia or hypodontia for physiological and psychosocial reasons.^8,9^

The successful use of any prosthesis is dependent on the cooperation and communication between the dental team and the patient and his parents. For example, great care will need to be taken to maintain the boy's oral hygiene in order to benefit from the long-term treatment plan. In addition, the boy and his parents must be educated and motivated about the dental problems related to his genetic and psychological conditions.^10,11^ Treatment of the ED patient generally includes a removable and/or fixed partial denture, a complete denture prosthesis, and an implant retained prosthesis when indicated.^12^ These treatment modalities can be used individually or in combination to provide an optimal result. The proper sequencing of treatment is important to achieve the desired function and esthetic results. Because of early age intervention and the need to frequently modify the intraoral prosthesis during rapid growth periods, a removable partial denture or complete denture prosthesis is indicated initially.^12^ Hypodontia is associated with lack of development of the alveolar ridge and results in less volume of bone for support of a conventional prosthesis. Therefore, implant supported prosthesis can be recommended for these patients, but xerostomia, poor oral hygiene and the lack of bone and also the growing age might cause problems in placing endosseous implants in growing children.^13,14^ The short-term survival data reported by Guckes et al^13,14^suggests that it is possible to successfully place dental implants in male and female patients of different ages with ED and congenitally missing teeth. However, they stated that a careful evaluation of each patient is necessary to determine the bone volume available for implant placement. Due to the girl's young age, poor oral hygiene, xerostomia and insufficient quantity of alveolar bone, endosseous implants placement was not possible. The bone height and width was not sufficient for implant insertion. Application of removable dentures may be the only restorative option for this patient.

## SUMMARY

Young patients with ED need to be evaluated early by a dental professional to determine the oral ramifications of the condition. When indicated, appropriate care needs to be rendered throughout the child's growth cycle to maintain oral functions as well as to address the esthetic needs of the patient. This clinical report demonstrates that removable cast partial dentures associated with direct composite restorations and overdenture can be a reversible and economical method of treatment for young ED patients.
